# K-Map: connecting kinases with therapeutics for drug repurposing and development

**DOI:** 10.1186/1479-7364-7-20

**Published:** 2013-09-23

**Authors:** Jihye Kim, Minjae Yoo, Jaewoo Kang, Aik Choon Tan

**Affiliations:** 1 Translational Bioinformatics and Cancer Systems Biology Laboratory, Division of Medical Oncology, Department of Medicine, University of Colorado Anschutz Medical Campus, Aurora, CO 80045, USA; 2 Data Mining and Information Systems Laboratory, Department of Computer Science and Engineering, Korea University, Seoul 136-713, South Korea

## Abstract

Protein kinases play important roles in regulating signal transduction in eukaryotic cells. Due to evolutionary conserved binding sites in the catalytic domain of the kinases, most inhibitors that target these sites promiscuously inhibit multiple kinases. Quantitative analysis can reveal complex and unexpected interactions between protein kinases and kinase inhibitors, providing opportunities for identifying multi-targeted inhibitors of specific diverse kinases for drug repurposing and development. We have developed K-Map—a novel and user-friendly web-based program that systematically connects a set of query kinases to kinase inhibitors based on quantitative profiles of the kinase inhibitor activities. Users can use K-Map to find kinase inhibitors for a set of query kinases (obtained from high-throughput ‘omics’ experiments) or to reveal new interactions between kinases and kinase inhibitors for rational drug combination studies.

**Availability and implementation:**

K-Map has been implemented in python scripting language and the website is freely available at: http://tanlab.ucdenver.edu/kMap.

## Introduction

Protein kinases represent one of the largest ‘druggable’ and well-studied families in the human genome [1]. This class of proteins (kinome) plays a key role as regulators and transducers of signaling in eukaryotic cells. There is an estimated >500 members of the human kinome which can be classified into seven different kinase families based on their conserved catalytic domain sequences [2]. Kinases are relatively easy to target with small molecules and have been extensively studied at the biochemical, structural, and physiological levels. In cancer cells, some kinases are mutated and acquire oncogenic properties to drive tumorgenesis. Small molecules that inhibit these oncogenic kinases can effectively kill cancer cells, as demonstrated by the success story of imatinib (Gleevec®, Novartis, Basel, Switzerland) in inhibiting the activity of *BCR-ABL* in chronic myelogenous leukemia [3]. Imatinib also inhibits *KIT* and *PDGFRA*, which are commonly dysregulated in gastrointestinal stromal tumors [4]. The imatinib example illustrates that small-molecule kinase inhibitors interact with multiple protein kinase family members (*BCR-ABL*, *KIT*, *PDGFRA*), and understanding these complex interactions between kinases and inhibitors could be useful for drug repurposing and development. These complex interactions could only be revealed by systematic interrogation of the small molecules across a large panel of kinases using quantitative assays (kinase activity profiles). Here, we have developed K-Map—a novel and user-friendly web-based program that systematically connects a set of query kinases to kinase inhibitors based on quantitative profiles of the kinase inhibitor activities. K-Map is motivated by the ‘connectivity map’ concept [5] where gene expression changes could be used as the ‘universal language’ to connect between biological systems, genes, and drugs. Instead of gene expression signatures, we used the kinase activity profiles as the ‘language’ for connecting kinases and small molecules in K-Map to reveal the complex interactions of kinases and inhibitors.

## K-Map methods and features

### Quantitative kinase inhibitor selectivity data sources

Two recently published comprehensive analyses of kinase inhibitor selectivity [6,7] were used to construct the K-Map reference database (kinase activity profiles). The first study systematically interrogates 178 commercially available inhibitors against a panel of 300 protein kinases using a radiometric phospho-transfer method to assess the percent kinase inhibition (IC_50_) [6]. The second study measures inhibitor selectivity and potency of 72 inhibitors across 442 kinases using direct binding affinities between inhibitors and kinases (K_d_) [7]. These kinase activity profiles were converted into rank-ordered lists according to their inhibitions and potencies against the kinases and used as the K-Map reference profiles for matching query kinases. For each study, the kinase activity profiles for individual drugs were converted into rank-ordered lists according to their inhibitions and potencies against the kinases. As a result, we generated two K-Map reference databases from these two studies: one for IC_50_ and the other one are for K_d_. Both databases will be used to connect the query kinases and return the drugs in K-Map.

### Pattern matching strategy

We implemented the K-Map pattern matching strategy based on the Kolmogorov-Smirnov (KS) statistics. The KS test is a nonparametric, rank-based pattern matching approach implemented in the connectivity map [5]. The query is a list of kinases, and the goal of the algorithm is to correlate kinase inhibitor that enriches the same kinases based on kinase inhibition profiles. For every inhibitor in the reference database, the KS statistic is computed and a connectivity score is defined.

Similar to the connectivity map approach [5], to compute the connectivity score, let *N* be the number of kinases in the reference database and *M* be the number of query kinases. For every drug in the reference database, we can compute the rank-ordered list *R* for all kinases (1, 2, …, *N*) based on the drug inhibitions and potencies against the kinases. For a list of query kinases of *j*, where *j* = 1, 2, …, *M*, compute the following two values for each drug *i* in the reference database:

$$a=\begin{array}{c} M\\ \max_{j=1} \end{array}\left[\frac{j}{M}-\frac{R\left(j\right)}{N}\right]$$

and

$$b=\begin{array}{c} M\\ \max_{j=1} \end{array}\left[\frac{R\left(j\right)}{N}-\frac{\left(j-1\right)}{M}\right]$$

Let *KS*^*i*^ be the KS score for drug *i*,

$$KS^{i}=\left\{\begin{array}{c} \begin{array}{cc} a, & \mathrm{if}\;a>b; \end{array}\\ \begin{array}{cc} -b, & \mathrm{if}\;b>a. \end{array} \end{array}\right)$$

Finally, to compute the connectivity score (*S*^*i*^) for drug *i* in a reference database, let *P* = max(*KS*^*i*^) and *Q* = min(*KS*^*i*^),

$$S^{i}=\left\{\begin{array}{c} \begin{array}{cc} \frac{KS^{i}}{P}, & \mathrm{if}\;KS^{i}>0; \end{array}\\ \begin{array}{cc} -\left(\frac{KS^{i}}{Q}\right), & \mathrm{if}\;KS^{i}<0. \end{array} \end{array}\right)$$

The connectivity score (*S*^*i*^) for every drug is reported as the ‘Score’ in the results page. A positive score represents that the inhibitor has a similar rank order as the query kinases, indicating that the inhibitor is more specific in inhibiting the query kinases. A negative score represents that the inhibitor has a reverse rank order as the query, hence not specific in inhibiting the query kinases. Connectivity scores for each inhibitor were normalized to yield a score ranging 0 to 1, and inhibitors were ranked based on this normalized score. We also computed the running sum of the connectivity score for each inhibitor. The maximum value of the running sum is equivalent to the connectivity score of each inhibitor. Since the query kinases are unitless, K-Map can be applied to any technology platform.

### Computing the permutation *p* value

To estimate the *p* value for each drug *i*, we perform a permutation test by randomly selecting the number of *M* instances from the rank-ordered drug *i* kinase profiles. Let *t* = 1, 2, …, *T* trials; the same procedure as computing the *KS*^*i*^ for drug *i* is performed *T* times and is denoted as $KS_{t}^{i}$. Let $KS_{0}^{i}$ denote the actual *KS*^*i*^ for drug *i*. Count the number of times *f* where

$${\left[\left|KS_{t}^{i}\right|\geq \left|KS_{0}^{i}\right|\right]}_{t=1}^{T}$$

is true. The frequency of this event (*f*/*T*) is estimated as a (two-sided) *p* value. This procedure is similar to the implementation of permutation test by the connectivity map [5]. The *p* value reported in the results page of K-Map is computed by 500 permutations.

### Query features

**Figure 1:**
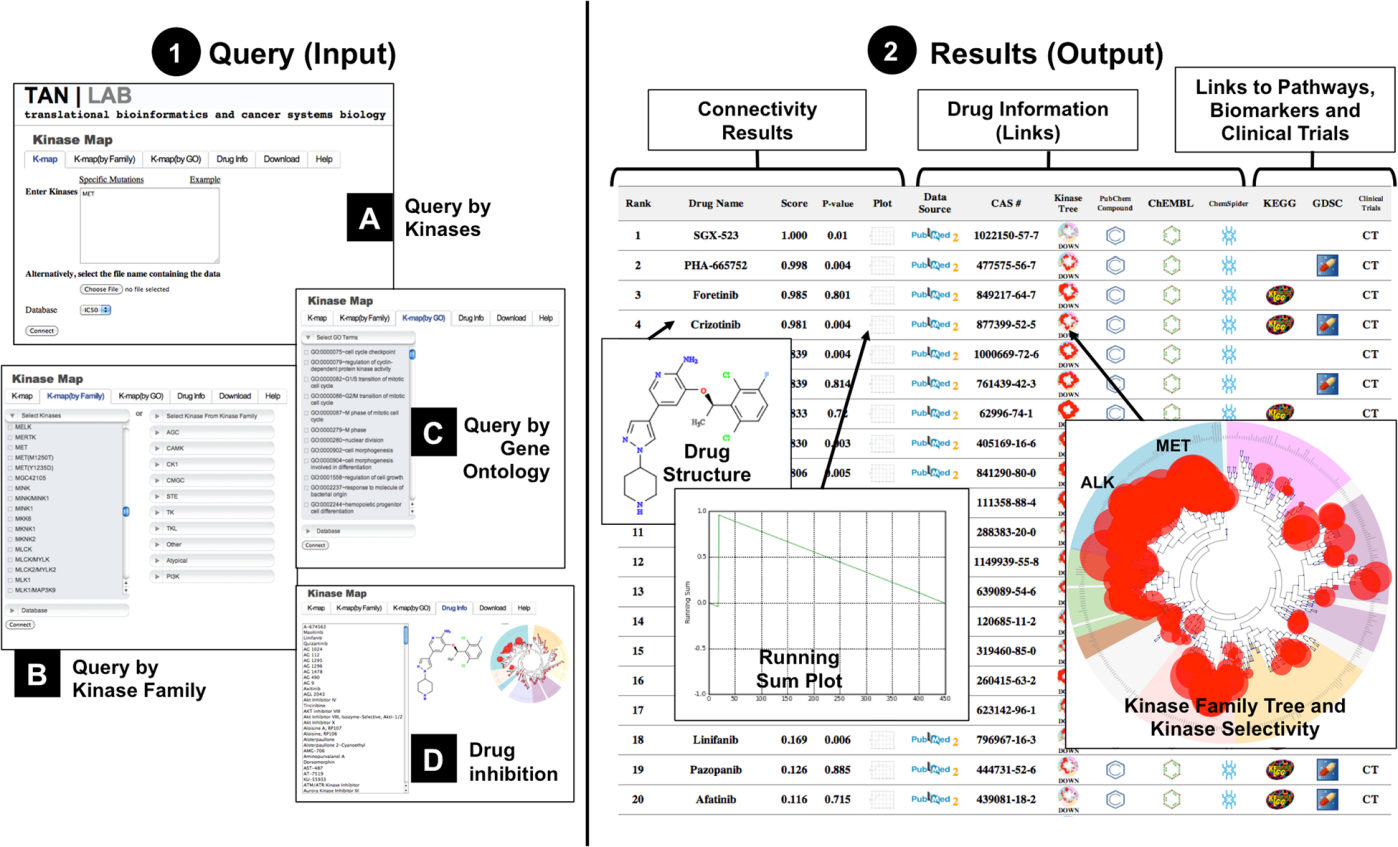
**Query and results of the K-Map.** Left, query features. K-Map could be queried by: **(A)** direct input or directly uploading a list of kinases in the K-Map tab, **(B)** by selecting kinases from the kinase family, or **(C)** querying by Gene Ontology Biological Processes. All drug information is available in the Drug Info tab **(D)** and could be downloaded in the Download tab. Right, connectivity results. Query kinases were connected to the inhibitors and sorted by normalized connectivity scores. Link-out features include PubMed, PubChem, ChEMBL, and ChemSpider for drug sources and information. K-Map also provides links to pathway, biomarker, and clinical trial information for each drug via KEGG, GDSC, and ClinicalTrials.gov databases, respectively.

K-Map implements three query functions: users can (1) directly enter query kinases in the query text box or upload a list of query kinases (Figure 1 (A)) in the K-Map tab, (2) select kinases from the kinase family (Figure 1 (B)) in the K-Map (by family) tab, or (3) query a set of kinases involved in certain biological processes according to Gene Ontology (Figure 1 (C)) in the K-Map (by GO) tab. Users also need to define which database they would like their query kinases to connect with (IC_50_ or K_d_). All inhibitors available in the K-Map could be browsed under the Drug Info tab (Figure 1 (D)). Under the Download tab, users can search and download kinase-inhibitor relationships. The Help tab provides user guide to query and navigate the K-Map. The user manual for K-Map is available at http://tanlab.ucdenver.edu/kMap.

### Connectivity results and linking features

The output of K-Map is a rank-ordered list of inhibitors based on the normalized connectivity scores, accompanied by *p* values and running sum plots. The 2D drug structure is viewable by scrolling through the drug name. Kinase inhibitor specificity within the kinase family tree is generated under KinaseTree column where the red circles indicate degrees of inhibition. Linking features are available for data source of the kinase inhibition assay (via PubMed) and three major chemical databases (PubChem [8], ChEMBL [9], and ChemSpider (http://www.chemspider.com)). Additional links to drug pathway and drug biomarkers are available through the Kyoto Encyclopedia of Genes and Genomes (KEGG) [10] and Genomics of Drug Sensitivity in Cancer (GDSC) [11] databases, respectively. K-Map also provides link-out to ClinicalTrials.gov for ongoing or completed clinical trials of these inhibitors in various diseases. We plan to update the K-Map database every quarter to keep up with the new data and link-out information.

### Implementation

K-Map is implemented in python (v2.6) and CGI script. The kinase family tree map and 2D drug structure are generated by the E.T.E. software (v2.0) and Open Babel (v2.3.1) [12], respectively. The K-Map website is freely available at: http://tanlab.ucdenver.edu/kMap.

### K-Map application: case study

**Figure 2:**
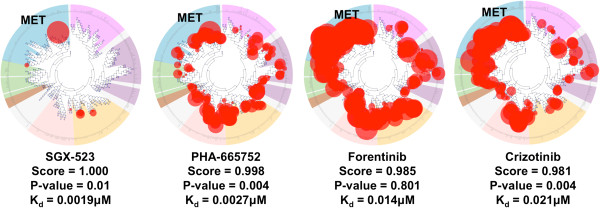
**Query results for *MET *using K**_**d **_**database.** These four compounds were returned with connectivity score >0.9, indicating that these compounds were highly specific in inhibiting *MET*. SGX-523 is ranked #1 and is a specific *MET* inhibitor, with K_d_ = 0.0019 μM as illustrated in the kinase family tree. PHA-665752 is ranked #2 with K_d_ = 0.0027 μM against *MET*. These two compounds (SGX-523 and PHA-665752) were preclinical compounds and have not moved into clinical trials. Forentinib is an FDA-approved drug that inhibits *MET* and *KDR* (K_d_ = 0.014 μM against *MET*) and is ranked #3; however, the *p* value is high (*p* = 0.801). Crizotinib is also an FDA-approved drug that inhibits *ALK*, and this compound has K_d_ = 0.021 μM against *MET* (*p* value = 0.004). Crizotinib was validated in *in vitro* experiments and showed synergistic effects when combined with Nutlin-3 in p53wild-type cancer cell lines [13].

We have recently performed a genome-wide functional genetic screen to identify synthetic lethality genes for Nutlin-3 (p53 inhibitor) in p53 wild-type cancer cell lines [13]. From this screening, we identified *MET* as a synthetic lethal gene with Nutlin-3 in killing cancer cell. By querying *MET* in the K-Map using K_d_ database (Figure 1 (A)), four compounds were returned with connectivity score >0.9 (Figure 1,right side). All four compounds are specific in inhibiting *MET* with K_d_ ≤ 0.025 μM (Figure 2). Interestingly, crizotinib, a recently FDA-approved *ALK* inhibitor is ranked #4 (*p* value = 0.004). As we demonstrated, treating p53 wild-type cancer cells with Nutlin-3 and crizotinib inhibits proliferation and enhances cell killing in *in vitro* experiments [13]. This supports the finding that the K-Map could reveal new inhibition of kinase inhibitor (Figure 1, kinase family tree indicates that crizotinib shows the highest selectivity in inhibiting *ALK* and *MET*).

### Summary

K-Map is a novel and user-friendly web-based tool for connecting kinases with drugs based on quantitative profiles of the kinase inhibitor activities. Many kinase inhibitors could promiscuously inhibit multiple kinases due to conserved sequence similarity among kinase family members; we have exploited these complex and unexpected interactions between kinases and inhibitors as opportunities for drug repurposing and development. Users can use K-Map to search kinase inhibitors for a set of query kinases (obtained from high-throughput ‘omics’ experiments) or to reveal new interactions between kinases and kinase inhibitors for rational combination studies. In the future, we plan to extend K-Map by including more kinase inhibitor profiles. In summary, we believe that K-Map will be a valuable bioinformatics tool in connecting altered/mutated genes identified by next-generation sequencing with therapeutics, accelerating the process of personalized medicine.

## Competing interests

The authors declare that they have no competing interests.

## Authors’ contributions

The authors wish it to be known that, in their opinion, the first two authors should be regarded as joint first authors. JK carried out the K-map studies, participated in the method implementation, and drafted the manuscript. MY prepared the dataset and implemented all web version of K-map. JK participated in the design of the study. ACT conceived of the study, participated in its design and coordination, and wrote the final manuscript. All authors read and approved the final manuscript.
